# Conceptual and empirical advances in Neotropical biodiversity research

**DOI:** 10.7717/peerj.5644

**Published:** 2018-10-04

**Authors:** Alexandre Antonelli, María Ariza, James Albert, Tobias Andermann, Josué Azevedo, Christine Bacon, Søren Faurby, Thais Guedes, Carina Hoorn, Lúcia G. Lohmann, Pável Matos-Maraví, Camila D. Ritter, Isabel Sanmartín, Daniele Silvestro, Marcelo Tejedor, Hans ter Steege, Hanna Tuomisto, Fernanda P. Werneck, Alexander Zizka, Scott V. Edwards

**Affiliations:** 1 Department of Biological and Environmental Sciences, University of Gothenburg, Gothenburg, Sweden; 2 Gothenburg Global Biodiversity Centre, Gothenburg, Sweden; 3 Gothenburg Botanical Garden, Gothenburg, Sweden; 4 Department of Organismic Biology and Evolutionary Biology, Museum of Comparative Zoology, Harvard University, Cambridge, MA, USA; 5 Laboratory Ecologie et Biologie des Interactions, Team “Ecologie, Evolution, Symbiose”, Université de Poitiers, Poitiers, France; 6 Department of Biology, University of Louisiana at Lafayette, Lafayette, LA, USA; 7 Federal University of São Paulo, Diadema, Brazil; 8 Museum of Zoology, University of São Paulo, São Paulo, Brazil; 9 Institute for Biodiversity and Ecosystem Dynamics, University of Amsterdam, Amsterdam, Netherlands; 10 Universidad Regional Amazonica IKIAM, Napo, Ecuador; 11 Instituto de Biociências, Departamento de Botânica, Universidade de São Paulo, São Paulo, Brazil; 12 Integrative Biology, University of California, Berkeley, CA, USA; 13 Real Jardin Botanico, Madrid, Spain; 14 Department of Computational Biology, University of Lausanne, Lausanne, Switzerland; 15 Swiss Institute of Bioinformatics, Lausanne, Switzerland; 16 Instituto Patagónico de Geología y Paleontología, Puerto Madryn, Guatemala; 17 Naturalis Biodiversity Center, Leiden, Netherlands; 18 Systems Ecology, Free University, Amsterdam, Netherlands; 19 Department of Biology, University of Turku, Turku, Finland; 20 Instituto Nacional de Pesquisas da Amazônia, Manaus, Brazil; 21 Gothenburg Centre for Advanced Studies in Science and Technology, Chalmers University of Technology and University of Gothenburg, Gothenburg, Sweden

**Keywords:** Biogeography, Biotic diversification, Landscape evolution, Phylogeny, Scale, Biodiversity, Community ecology, Phylogeography, Phylogenetics, Tropics

## Abstract

The unparalleled biodiversity found in the American tropics (the Neotropics) has attracted the attention of naturalists for centuries. Despite major advances in recent years in our understanding of the origin and diversification of many Neotropical taxa and biotic regions, many questions remain to be answered. Additional biological and geological data are still needed, as well as methodological advances that are capable of bridging these research fields. In this review, aimed primarily at advanced students and early-career scientists, we introduce the concept of “trans-disciplinary biogeography,” which refers to the integration of data from multiple areas of research in biology (e.g., community ecology, phylogeography, systematics, historical biogeography) and Earth and the physical sciences (e.g., geology, climatology, palaeontology), as a means to reconstruct the giant puzzle of Neotropical biodiversity and evolution in space and time. We caution against extrapolating results derived from the study of one or a few taxa to convey general scenarios of Neotropical evolution and landscape formation. We urge more coordination and integration of data and ideas among disciplines, transcending their traditional boundaries, as a basis for advancing tomorrow’s ground-breaking research. Our review highlights the great opportunities for studying the Neotropical biota to understand the evolution of life.

## Introduction

**Figure 1:**
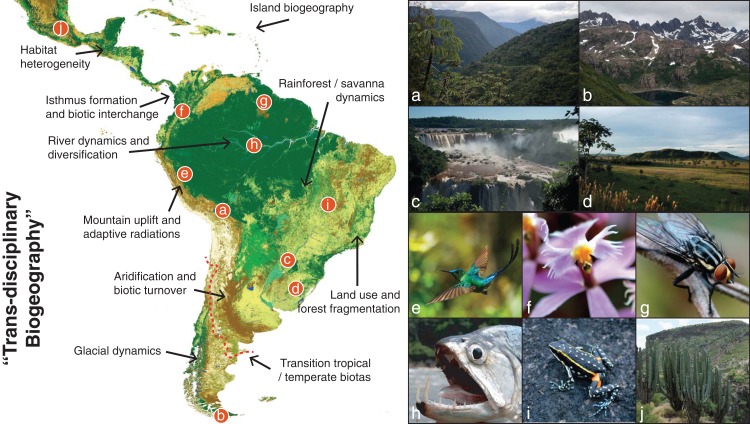
The giant Neotropical puzzle. Map of the Neotropical region, spanning from Central Mexico to central Argentina (red dashed line) and including all Caribbean Islands. The figure shows examples of the large diversity of Neotropical habitats and the taxa that inhabit those habitats. We also outline a few of the many topics in Neotropical biodiversity that can be studied in the “trans-disciplinary biogeographic approach” advocated here. (A) Eastern slopes of the Bolivian Andes, where the Amazonian and Andean biotas meet; (B) Patagonian mountains of southern Chile, which despite being in the temperate zone of South America is home to many Neotropical-derived lineages; (C) Iguazu waterfalls, where increased humidity create gallery forests within the South American open diagonal; (D) Southern grasslands of the Pampas, a naturally open habitat now largely influenced by human activity; (E) One of the ca. 338 known species of hummingbirds, a conspicuous clade currently restricted to the American continent and particularly diverse in the Andes; (F) *Epidendrum ibaguense*, a widespread species in the orchid family in which many new Neotropical species are discovered each year; (G) An unidentified fly in the inselbergs of southern French Guiana, where basaltic rocks emerge several hundred meters above the surrounding Amazonian rainforest; (H) The large dogtooth characin fish *Hydrolycus scomberoides*, exemplifying the world’s richest ichthyofauna in the Amazon drainage basin; (I) *Ameerega flavopicta*, a rock-dwelling frog species adapted to a region of high seasonality of precipitation; (J) A columnar cactus of central Mexico, near the northwestern limits of the Neotropical region where low-canopy forests and succulent vegetation build vegetation mosaics across the landscape. Map generated through the remote-sensing ESA GlobCover 2009 project and colored by biome assignments (©ESA 2010 and UCLouvain; http://due.esrin.esa.int/page_globcover.php). Photo credits: A–G, I and J: A.A.; H: J.A.

The Neotropical region (also referred to as tropical America or the American tropics) extends today from central Mexico to Argentina, including the Caribbean (Morrone, 2013). The region encompasses a vast range of biomes and habitat types, each with a particular history of landscapes and biotic evolution (Fig. 1; Hughes, Pennington & Antonelli, 2013). For many groups of organisms, the Neotropics are home to outstandingly high levels of species richness, when compared to other major biotic realms (Lundberg et al., 2000; Antonelli & Sanmartín, 2011). As such, understanding Neotropical biodiversity patterns and the processes associated with its origin and maintenance represents a major scientific priority.

Biodiversity refers to the diversity of life across all levels of biological organization (Purvis & Hector, 2000; Gaston & Spicer, 2004). Biodiversity is unevenly distributed across Earth, and varies among and within geographic regions, between terrestrial and aquatic ecosystems, and among different groups of organisms. Biodiversity increases from the poles to the equator, reaching the highest values in tropical regions, a pattern termed the latitudinal diversity gradient (Willig, Kaufman & Stevens, 2003; Field et al., 2009). However, this pattern is complex, with numerous non-diverse tropical or diverse non-tropical areas and taxa constituting exceptions. In addition, there are still numerous uncertainties in the underlying data used to generalize overall patterns. Most importantly, these patterns remain far from properly understood, and we are still struggling to identify their main determinants. As a result, researchers tend to focus on different aspects of biodiversity such as taxonomic, phylogenetic, and functional diversity (FD) (Swenson, 2011; Tables 1–3). Each of these aspects of biodiversity may vary among regions and taxa, and each must therefore be assessed by independent criteria (Strecker et al., 2011; Magurran, 2013).

**Table 1:** The concept of taxonomic diversity, and its use and challenges in the Neotropical context.

**Definition.** Taxonomic diversity refers to how many taxa can be found within a given area or higher clade, and how individuals are distributed among these taxa. Taxonomic diversity can be quantified at different taxonomic ranks (e.g., species, genera, families), with the species rank being the most popular by far. Species richness is widely viewed as a fundamental measure of overall biodiversity (Gotelli & Colwell, 2001). This is due to the fact that the species boundary defines the limits of genetic variation, natural selection, and adaptation (Sexton et al., 2009). While individual organisms live and die, the stable phenotypes recognized as species may persist for millions of years, serving as predictable components of the ecosystems in which all species function and evolve (Eldredge, 1989). As result, species are thought to constitute the basic structural and functional units in ecology and evolution (Tilman & Downing, 1994; Worm et al., 2006).
Generic and family-level taxonomic ranks are occasionally used in comparative studies, especially when species identification or delimitation is difficult (Bertrand, Pleijel & Rouse, 2006). However, the ranks that taxonomists must assign to higher-level taxa are often considered to be arbitrary constructs, reflecting little biological organization, and incorporating further biases and artefacts when compared, although opposing views exist (Humphreys & Barraclough, 2014). In general, species are seen as the “fundamental category of biological organization” despite the multitude of species definitions available (De Queiroz, 2005).
**Metrics and usage.** Taxonomic diversity is most commonly measured using taxon richness, that is, the number of taxa in a given area. However, relative abundance distributions can differ greatly among areas, and an area where taxon abundances are equal has intuitively higher diversity than an area with the same number of taxa but a high degree of dominance by one or a few taxa. Abundance differences can be taken into account by quantifying diversity as the effective number of species (also known as Hill number or true diversity; Hill, 1973; Jost, 2006; Tuomisto, 2010, 2018). Because its values are easier to interpret and compare than those of traditional diversity indices (e.g., Brillouin, Shannon, and Simpson indices), the effective number of species is emerging as the best general measure of diversity by a broad consensus. Quantitative abundance data are rather rare, though, and few studies have included abundance when discussing diversity in the Neotropics (but see Valdujo, Carnaval & Graham, 2013; Ter Steege et al., 2013; Tuomisto, Zuquim & Cárdenas, 2014; Jenkins et al., 2015; Moura et al., 2016; Azevedo, Valdujo & Nogueira, 2016; Gómez et al., 2018).
Observed taxonomic diversity is sensitive to sampling effort, especially at the species rank. Since communities typically contain many species that are locally rare, observed species richness provides only an underestimate of the number of species actually present, unless the community is very thoroughly sampled. The accuracy of estimates of taxonomic diversity depends on the number of individuals sampled, the size of the local species pool, the evenness of species abundances in the community, size and environmental heterogeneity of the area, and the status of taxonomic knowledge of the groups surveyed. When comparing estimates of local taxonomic diversity among areas, it is therefore important that they are based on quantitative and standardized sampling (Chao & Jost, 2012; Tuomisto, 2018).
Beta diversity and species turnover, reflecting heterogeneity in species composition among sites, are also of interest (Tuomisto, 2010, 2018; Higgins et al., 2011; Leprieur et al., 2011; J. M. Craig et al., 2018, unpublished data). However, quantifying these requires data where species identifications have been done consistently using a standard taxonomy, and such data are only available in some areas for some vascular plants (e.g., trees, ferns; Tuomisto, Ruokolainen & Yli-Halla, 2003; Arellano et al., 2016; Tuomisto et al., 2016), and some vertebrates (e.g., birds, primates, some fishes; Arrington & Winemiller, 2009). For these same organisms, a general understanding of species richness gradients has emerged (Kier et al., 2005; Albert et al., 2011; Rosauer & Jetz, 2014; Tuomisto, Zuquim & Cárdenas, 2014). For most other organisms, too few data are available to allow accurate circumscriptions of taxa and reasonable estimates of species richness gradients and species turnover (Andújar et al., 2015). Indeed, the smaller and less conspicuous the organism, the poorer the state of knowledge. For instance, very little is known about microbial and fungal diversity, and insect diversity is similarly understudied (Basset et al., 2012). However, even among well-studied and charismatic Neotropical taxa—such as birds and mammals, even river dolphins—there are still new species to discover (Hrbek et al., 2014).

**Table 2:** The concept of phylogenetic diversity, and its use and challenges in the Neotropical context.

**Definition.** Phylogenetic diversity (PD) assesses cumulative evolutionary distinctiveness within and among areas and taxa. We do not contest the usefulness of species as entities in the assessment of biodiversity patterns, conservation, and many other disciplines, from medicine to bioengineering. However, species are not universally comparable units, given differences in species concepts, operational criteria of delimitation, and circumscriptions among areas, taxa, and taxonomists. In addition, species differ widely in their evolutionary ages, geographic distributions, habitat tolerances, and degree of genetic structure. Species also differ in the biological attributes of their constituent organisms, and therefore, in the effects that these traits may have on ecological and evolutionary processes. Furthermore, species are really just the tips of larger phylogenetic trees evolving through time. PD is therefore a useful measure to directly compare the degree of phylogenetic divergence among groups and regions.
**Metrics and usage.** The basic idea of PD is to measure the total amount of lineage evolution through time (i.e., branch length) observed among all members of a clade or area (Faith, 1992). Overall, PD has been shown to provide a better estimate of “feature divergence” than species richness alone (Forest et al., 2007), because PD multiplies the species (tips of the tree) and multi-species clades (tree branches with multiple tips) by their phylogenetic age, usually measured in millions of years. However, there are many ways of deriving and applying such metrics from phylogenies. As such, researchers should try to choose the most appropriate index for each situation, as well as acknowledge these differences in cross-taxonomic comparisons (Tucker et al., 2016). Phylogenetic diversity (or more explicitly: divergence), although not “visible” constitutes a potentially powerful concept for increasing the standardization of biodiversity analyses, the recognition of areas for conservation, and our understanding of evolutionary history of clades, among others.
Complementary to phylogenetic diversity based on the relationships among taxa, patterns of genetic variation within species also represent a vital but often under-appreciated component of biodiversity. Knowledge of intraspecific genetic variation may also improve the prediction of a species ability to adapt to changing climates, as well as improving understanding of the process of speciation. This type of information is particularly important in the light of ongoing anthropogenic climate change. However, our current knowledge of species genetic diversity is restricted to a few selected species, and overall patterns of intraspecific genetic diversity remain poorly understood. Even among well-studied groups (e.g., mammals), spatial patterns of genetic diversity are effectively unknown within the tropics.

**Table 3:** The concept of functional diversity, and its use and challenges in the Neotropical context.

**Definition.** Functional diversity (FD) measures differences in the physiological, behavioral, and ecological characteristics of organisms, and how biological trait values (such as body mass of animals, and life form or habit of plants) affect ecological and evolutionary processes. Knowledge about species traits and ecological functions (such as a species trophic level, and including the variation in traits within and among species) is a crucial component of biodiversity. However, this is one of the major shortcomings in current biodiversity knowledge, especially in tropical areas. Few studies to date have mapped large-scale patterns of functional diversity, although efforts in this direction are underway (see for fishes e.g., Arbour & López-Fernández, 2014; Toussaint et al., 2016).
**Metrics and usage.** Apart from the lack of data, the theory behind functional diversity is not yet well consolidated. We still do not know which traits are ecologically and evolutionarily important for different groups, how to compare traits for different sets of organisms, and how functional diversity affects ecosystem productivity, stability, and resilience, especially in the tropics. An additional shortcoming is associated with biotic interactions. Apart from basic information on pollination and dispersal syndromes, we know surprisingly little about most biotic interactions. Very few species interaction networks are available to date (Toju et al., 2017).

## Survey Methodology

In April 2017, we gathered scholars from several countries and scientific backgrounds to discuss Neotropical biodiversity during the “Origins of Biodiversity Workshop” organized by Chalmers University of Technology and the University of Gothenburg (Sweden), under the auspices of the Gothenburg Centre for Advanced Studies. We spent one week outlining the topics presented here, focusing primarily on recent advances and the future of the field. We continued to work remotely toward the conclusion of this publication. The overall goal of this review is to summarize the knowns and unknowns about Neotropical biodiversity, with focus on terrestrial taxa and ecosystems, and to discuss the many opportunities and challenges of this research field. We acknowledge that the wide breadth of the topics discussed here are in part due to this being a summary of ideas produced at a workshop of diverse participants, and that each topic contains a depth that cannot be simply synthesized. For instance, more comprehensive reviews on the theories proposed to explain the origin of Neotropical diversity can be found elsewhere (Moritz et al., 2000; Antonelli & Sanmartín, 2011; Leite & Rogers, 2013; Table 4). We focus here on topics that we think require further development within this research field.

**Table 4:** Some of the many theories proposed to explain the high levels of (Neo)tropical biodiversity.

Theory	Key proponent(s)	Summary	Comment
Riverine barrier hypothesis	Wallace (1889)	The formation of large lowland Neotropical rivers like the Amazon resulted in genetic isolation and speciation in taxa ecophysiologically restricted to non-flooded rain forests.	Dynamic river capture is even more effective at isolating and reuniting populations than is the static geometry of dendritic river basins.
Pleistocene refugium theory	Haffer (1969)	Most Amazonian birds, and probably other taxa, originated recently in response to Pleistocene climate changes. The repeated contraction of forests in relation to savannas led to the isolation of populations and inability of breeding once they came into secondary contact during inter-glacials.	From initial support for plants and other taxa, this theory has been heavily criticized based on lack of geophysical evidence for savanna expansions, old divergence times from phylogenies, etc.
Time-area integrated hypothesis	Fine & Ree (2006)	Diversity can be predicted by the amount of time that species spend in a region, multiplied by the total area of that region.	A modification of this model is a strong predictor of dispersal events across the Neotropics (Antonelli et al., 2018).
Phylogenetic niche conservatism	Wiens & Donoghue (2004)	Tropical biotas are more diverse because many lineages of the modern biosphere evolved in the super-greenhouse world of the Mesozoic and early Cenozoic 140–50 Ma and remained in their original environment.	Most clades have origins in warm and wet tropical climates. Most clades at higher latitudes adapted to cold and dry conditions in the Neogene and Quaternary.
Out of the Tropics	Jablonski, Roy & Valentine (2006)	Tropical biotas are more diverse because lineages have higher speciation rates, lower extinction rates, and higher net emigration over immigration than lineages in extra-tropical regions.	This is just one popular theory among several others attempting to explain the latitudinal diversity gradient in species richness.
Metabolic theory of ecology	Brown & Svenning (2013)	Higher metabolic rates translate into higher rates of speciation and extinction at low latitudes.	Incompletely developed mechanistic links between kinetics at the metabolic, ecophysiological, and evolutionary scales.
Tropical productivity	Gillman et al. (2014)	Species richness is positively correlated with net primary productivity because larger populations are less likely to stochastically fluctuate to a population size of zero, which is a sticky boundary.	Metanalyses have shown a unimodal relationship is more common than a monotonic between productivity and species richness (Fraser et al., 2015).
Sea‐level fluctuations	Nores (2002)	Repeated sea-level rises during the late Cenozoic led to the allopatric speciation of Amazonian species in true islands.	Model based on current topography, lacking other geophysical evidence.
Museum hypothesis	Stebbins (1974)	Tropical lowlands act as “museums” of diversity, in which species of different origins gradually accumulate.	The Neotropics is now considered both “museum” and “cradle” of diversity (McKenna & Farrell, 2006).

## What Do We Know About Patterns of Neotropical Biodiversity?

### Taxonomic diversity

**Figure 2:**
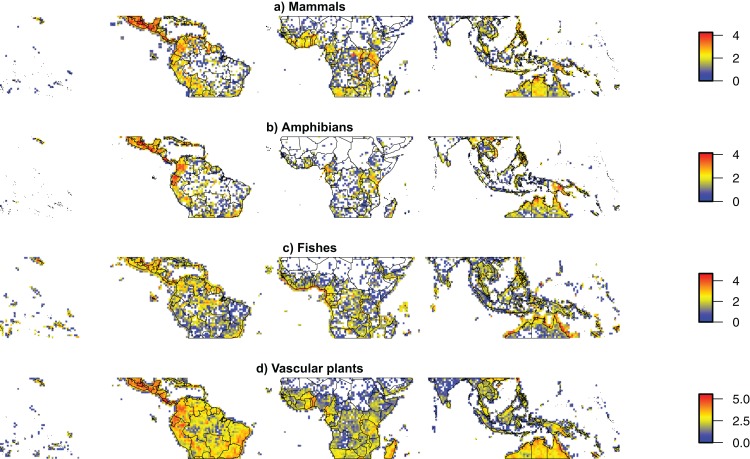
Taxonomic sampling across the world’s tropics. Density maps for geo-referenced species occurrences available from the Global Biodiversity Information Facility for (A) Mammals, (B) Amphibians, (C) Fishes, (D) Vascular plants between the Tropics of Cancer and Capricorn (23.5°S–23.5°N), showing the main spatial biases of taxonomic sampling. All datasets were cleaned for automatically detectable errors using SpeciesGeoCoder (Töpel et al., 2016). The figure is shown on a cylindrical equal area projection with standard parallels of 11.75°S and 11.75°N. The width of each cell is consistently 1°, while the height of each cell is 1° at the standard parallels, slightly lower at the equator and slightly higher at the Tropics of Cancer and Capricorn. Colors indicate 10-based logarithm of the number of records.

Relatively good estimates of taxonomic diversity are only available for well-studied Neotropical taxa, as in other parts of the world. These estimates have been used to identify the best predictors of diversity at large scales (Jenkins et al., 2015; Moura et al., 2016). Although sampling across taxa is comparable or even greater in the Neotropics than in other tropical regions (Fig. 2; Table 1), taxonomic diversity is generally underestimated within the Neotropics, especially for poorly sampled organisms such as fungi, invertebrates, and micro-organisms.

Several examples of species-rich, yet incompletely-documented faunas are available, including large clades of freshwater fishes, amphibians, and some groups of reptiles. Although about 5,600 species of freshwater fishes are currently known in the Amazon, the Orinoco, and adjacent river basins of tropical South and Central America, more than 100 new species are described every year (Van Der Sleen & Albert, 2017). In other words, approximately two new species are described per week, although an even higher number of new species would be expected if more trained taxonomists were available. This rapid pace of species description is not slowing down, and recent estimates for the total number of Neotropical freshwater fishes exceed 8,000 species (Reis et al., 2016). This estimate is remarkable, implying that more than 2,400 fish species might remain to be described in the Neotropics alone, a number that exceeds the combined number of rodent species currently known on Earth. This large number of expected, but still hidden, lineages represents an example of the “known unknowns” of Neotropical biodiversity (Table 5), which may have different underlying explanations (such as lack of taxonomic training, uneven distribution of resources across taxa and habitats, morphological complexity, among others).

**Table 5:** The various components of Neotropical biodiversity, examples of major aspects known about them, and some of the key topics that remain to be understood.

Biodiversity components	Known knowns	Known unknowns	Unknown unknowns
Taxonomic diversity	Approximate species numbers for macroscopic organisms; human impacts tend to decrease overall diversity	Large portions of biodiversity are unexplored (i.e., microbes, invertebrates, fungi)	Taxonomic units used in biodiversity studies may not represent comparable ecological or evolutionary units
Genetic variation (within species)	Patterns of genetic variation known for very selected taxa	Overall patterns of genetic variation	How generalizable are conclusions drawn by such limited patterns of genetic diversity
Phylogenetic diversity	General understanding of the tree (or network) of life	Drivers of diversification	Potential biases in phylogeny reconstruction and time-calibration
Spatial patterns of diversity	Hotspots and general patterns of species richness and diversity; broad species ranges for charismatic taxa	Areas of endemism; known patterns of biodiversity are biased; ecological preferences of species; drivers of diversity	Human impact to overall spatial patterns
Functional diversity	Large scale productivity patterns	Biotic interactions	Relevance of current functional diversity measures; equivalency in functional traits; relationship between current and future functional diversity

Current knowledge of taxonomic limits of Neotropical amphibians and reptiles is gradually improving. Several molecular studies have detected high levels of cryptic diversity, that is, the existence of two or more lineages within a known species (Bickford et al., 2007), indicating that the known taxonomic diversity is still underestimated in many orders (Funk, Caminer & Ron, 2011; Fouquet et al., 2012). Even in the much more densely sampled and well-studied Atlantic Rainforest of Brazil, charismatic species of frogs are still being discovered. For instance, seven new species of *Brachycephalus* were recently described for this region (Ribeiro et al., 2015). Likewise, intraspecific analyses of Neotropical lizards show that the occurrence of cryptic diversity is often manifested across biomes. This subdivision of broadly distributed taxa into multiple cryptic species with more restricted geographic distributions increases the perception of biological diversity of a given region, and has numerous implications for biogeography (Werneck et al., 2012b) and conservation (Simões et al., 2014).

For plants, a quantitative assessment on the discovery of Amazonian trees during the last three centuries was compiled by Ter Steege et al. (2016), showing clear peaks in herbarium collections and new species descriptions. Although the data show a drop in the collection of unknown taxa after the 1980s (Ter Steege et al., 2016), there are still enormous discoveries to be made. For example, in a few years of increased collection efforts, the *Guide of the Ducke Reserve* (Brazil; Ribeiro, 1999), which covers one of the most thoroughly studied areas of Amazonian forest, increased the number of known vascular plants from 825 (Prance, 1990) to 2,079 (Hopkins, 2005).

One difficulty in assessing taxonomic diversity is that taxonomic units may vary according to the preference of the taxonomist revising a particular group (e.g., whether a “splitter” or a “lumper”), and by the data and methodologies underlying taxonomic revisions and species circumscriptions. This issue becomes obvious when taxonomic treatments of the same group are produced by different researchers independently. For example, the Neotropical palm genus *Attalea* included 29 species in one monograph (Henderson, Galeano & Bernal, 1995), and 65 species in another taxonomic treatment published just 4 years later (Glassman, 1999). Similarly, the Caribbean palm genus *Coccothrinax* included 14 species in one taxonomic treatment (Henderson, Galeano & Bernal, 1997) and 53 species in another (Dransfield et al., 2008). Personal preferences to “lumping” vs. “splitting” among taxonomists may have large consequences for biodiversity estimates and have been shown to strongly affect diversification rate estimates (Faurby, Eiserhardt & Svenning, 2016). Taxonomic practices should therefore be considered when comparing taxonomic biodiversity at any scale, and whenever adequate, researchers should take advantage of explicit and reproducible criteria for species delimitations.

Besides lumping vs. splitting, species lists may vary among authorities depending on the inclusion criteria, such as whether or not to include rare occurrences of a species common elsewhere, and how to classify the life forms of species (e.g., primarily herbaceous plants rarely recorded as trees). For large regions such as Amazonia, these are some of the reasons why the number and contents of species lists may differ substantially (Cardoso et al., 2017; H. Ter Steege et al., 2018, unpublished data).

### Phylogenetic diversity

Many Neotropical clades are known from just one or a few species that may represent relictual survivors of ancient and otherwise extinct groups (Table 2). This phenomenon is known from most organism groups, from Neotropical fishes (Albert et al., 2011) to plants (Wilson et al., 2012). To study how differences in diversity arise among taxa, some researchers have turned their attention to the study of early-branching, low-diversity clades. Examples of such clades include the leafy cacti (*Pereskia* and *Leuenbergeria* spp.; Cactaceae), the South American lungfish (*Lepidosiren paradoxa*; Lepidosirenidae), the hoatzin (*Opisthocomus hoazin*; Opisthocomidae), and the coral pipe snake (*Anilius scytale*; Aniliidae). In contrast, other species are members of species-rich Neotropical clades still in the full bloom of their diversification, like the lianas of tribe Bignonieae with more than 400 species (Lohmann & Taylor, 2014), palms with over 730 species (Dransfield et al., 2008), armoured catfishes (Loricariidae) with 680 species (Armbruster, Van Der Sleen & Lujan, 2018), and tanagers (Thraupidae) with 371 species (Burns, Unitt & Mason, 2016).

The first attempts to map phylogenetic diversity (PD) over continental and global scales were conducted for select vertebrate groups for which phylogenies were available and for which distribution patterns are relatively well known, such as amphibians, birds, and mammals (Safi et al., 2011). Other than these, large-scale phylogenetic and FD studies with focus and dense sampling in the Neotropics are scarce. Some progress has been made in mapping PD patterns in the Neotropics for specific clades (Lehtonen et al., 2015; Lovejoy et al., 2010; Rossatto, 2014; Fenker et al., 2014) or at the intraspecific level in the search for areas of high phylogeographic diversity and endemism (Carnaval et al., 2014; Smith et al., 2017; Melo et al., 2018). Several ongoing studies by independent research groups are now working to broaden our knowledge on the spatial distribution of Neotropical PD.

## Biases and Gaps in Neotropical Biodiversity Knowledge

There are two main sorts of biodiversity biases and gaps: taxonomic (also called the “Linnaean shortfall”) and spatial (the “Wallacean shortfall”). Taxonomically, detailed information on the richness and geographical distribution of species is restricted to certain well-studied taxa (e.g., primates and birds). We also know much more about organisms on land than those living in freshwater systems, including lakes, rivers, and swamps, leaving a large gap in the knowledge of Neotropical aquatic diversity. Spatially, our knowledge is concentrated to few well-studied areas (e.g., La Selva in Costa Rica, Barro Colorado Island in Panama, the Ducke Reserve in Brazil, Manu National Park in Peru, Yasuni National Park in Ecuador). Some regions stand out as having the lowest levels of sampling, including some parts of Central America, the central Andes, the Caatinga, and large parts of Amazonia (Fig. 2), where we have almost no occurrence records available (Hopkins, 2005; Feeley, 2015; Ter Steege et al., 2016; Tedesco et al., 2017). Clearly, for the vast majority of taxa, regions, and ecosystems, biodiversity knowledge is still scarce.

In general, knowledge of species distributions and diversity patterns are strongly biased toward areas that are more easily accessible by roads, rivers, and research stations (Hopkins, 2005; Meyer et al., 2015). Interestingly, at least Amazonian trees, there seems also to exist a bias toward reporting rare species (Table 6), as most scientific collectors tend to over-collect rare or uncommon trees (Ter Steege et al., 2011), although this pattern may not exist for other taxa. Although bioinformatic solutions may now assist in cleaning, predicting, and validating species occurrence data, taxonomic expertise is still essential but limited (Maldonado et al., 2015; Töpel et al., 2016). As a result of our limited knowledge on species distributions patterns, and large gaps in knowledge about climatic and edaphic conditions for large portions of the Neotropics, the ecological requirements for most species remain unknown (Table 5).

**Table 6:** The commonness of rarity in Neotropical diversity.

Most Neotropical species are rare, narrowly distributed, and endemic to particular regions or biomes (see Albert et al., 2011 for fishes; Ter Steege et al., 2016 for plants). Species with low abundances and narrow geographic ranges, as well as those confined to special habitats or areas, represent a sizable portion of Neotropical diversity. Indeed, a recent study extrapolating population size for Amazonian trees suggests that most species in the region are represented by comparatively few individuals (Ter Steege et al., 2013). Another study suggests that a considerable fraction of the rare species in the region may actually have relatively large distribution ranges (Zizka et al., 2017). However, many apparently widespread species in most taxonomic groups have been shown to contain multiple phylogenetic species, a possibility that remains poorly explored in the Neotropics but has important consequences for our understanding of diversity patterns and conservation priorities (Bickford et al., 2007; Colli et al., 2016). The contributions of rare species to diversity patterns are difficult to quantify and remain largely obscure (Coddington et al., 2009), partly because most truly rare species will be completely unknown, and partly because rareness in the ecological sense is hard to define, depending on a variety of aspects, including the species concept adopted and the taxonomic preferences.
In both fish and plant taxa, areas of endemism separated by prominent biogeographic barriers, such as Amazonian and Mesoamerican rainforests currently separated by the Andes, arise from dispersal limitation, and differential environmental tolerances (Bemmels et al., 2018). In contrast, for some tree genera of rainforest trees, dispersal does not seem to be a constraining factor, meaning that community assemblages either represent random draws from the possible species pools available (Dexter et al., 2017), or functional differences arising from different habitat tolerances. The geographic distributions of many riverine and floodplain taxa are limited by river basin watersheds, and opportunities for dispersal include river capture events (Albert et al., 2017). Finally, it is not enough to know where particular species occur; we also need to know where these species do not occur (Soria-Auza & Kessler, 2008). It is, therefore, difficult to reliably say if the biodiversity patterns known to date really reflect true patterns or biases in collection effort. Further, patterns of species richness are usually discerned relatively early in the documentation of a newly explored biota, whereas patterns of species endemism are more difficult to discern as they require positive knowledge of both where species are present and absent (Soria-Auza & Kessler, 2008).

## Introducing “trans-Disciplinary Biogeography”

Here, we propose that the best way to fully understand the complexity of Neotropical biodiversity is by conceptualizing and implementing a novel holistic framework. We define **trans-disciplinary biogeography** as “*a holistic framework that takes advantage of the methods and data in multiple disciplines, in order to solve complex questions about the evolution, maintenance, and distribution of biodiversity through time and scape. By doing so, each individual discipline transcends its traditional borders.”*

The idea and need of combining data from different sources in biogeography has been advocated before (e.g., Ribas et al., 2012; Weeks, Claramunt & Cracraft, 2016) but we propose a major expansion. Some examples of the constituent disciplines in this pursuit include biology (e.g., community ecology, phylogeography, systematics, taxonomy, historical biogeography; Lomolino, Riddle & Whitakker, 2017), geology (e.g., palaeontology, sedimentology, geomorphology), and climatology (e.g., modeling, speleology), amongst others. Successful examples of trans-disciplinary research include the archaeology-ecology synergy that led to the elucidation of pre-Columbian effects on the distribution of Amazonian plants (Levis et al., 2017); the genetic-geology synergy that led to the discovery of an earlier and more prolonged biotic interchange between South and North America since the Miocene (Bacon et al., 2015; De Baets, Antonelli & Donoghue, 2016), and the geology-biogeography-systematics synergy that led the discovery of a Miocene origin for the modern transcontinental Amazon river (Hoorn et al., 2010b; Albert, Val & Hoorn, in press). Some of these interactions are already recognized as new sub-disciplines, such as “community phylogenetics” (Swenson, 2011), “geogenomics” (Baker et al., 2014), and “geodiversity” (Gray, 2004). We envision the integration of a high number of additional synergistic sub-disciplines.

In practice, we want to encourage young students and researchers to invest time in learning more about disciplines that might fall outside their general curriculum, but which could contribute to creating fruitful synergies. Obviously, not every project or publication has to (or should) be trans-disciplinary, and this pursuit should not decrease the depth of a researcher’s skills in her or his topic of expertise. But without trans-disciplinary frameworks that are defined in early stages of new research projects, there is a risk that important perspectives are missed out.

To showcase the benefit of these interactions, we provide some background on the emergence of Neotropical biogeography as a research focus—which was integrative from its early days, but successively lost much of its cross-disciplinarity. We then discuss how trans-disciplinary biogeography may help address the interactions between landscape evolution, climate change and biotic diversification at its multiple levels (see also Hoorn, Perrigo & Antonelli, 2018).

### Early ideas about Neotropical biogeography

The Prussian naturalist Alexander von Humboldt was among the first to realize that biotic and abiotic processes interact to constrain species distributions, and to place these influences into a geological and climatic framework. He came to this notion in the Neotropics, most famously during his study of the Chimborazo volcano in Ecuador, where he carefully documented the location of different species along elevational zones (Humboldt & Bonpland, 1805). It was in this trip that he first observed that physical parameters such as topography and climate were key for geographic distributions.

A century later, Wegener (1912) advanced the incipient field of historical biogeography with the theory of continental drift, based in part on past geographic distributions of biotas linked by previously connected continental plates. The striking fit between the coastlines of South America and Africa was one of the pieces of evidence inspiring Wegener’s theory of dynamic, non-static landmasses. In the 1960s, a geophysical mechanism for plate tectonics was proposed (Vine & Matthews, 1963), placing studies of plant and freshwater fish biogeography into a plate tectonic framework, where vicariance was assumed as a major biogeographic force (Raven & Axelrod, 1974; Rosen, 1975).

At first, the explanatory power of vicariance biogeography was the ability to predict biogeographic distributions of individual taxa and that of whole biotas from knowledge of how landscapes changed through time (Rosen, 1978). The paradigmatic example is the geological fragmentation of the Gondwana supercontinent, and the resulting fragmentation of the resident Gondwanan biotas. The vicariance biogeography approach satisfies the scientific impulse of systematists and biogeographers for general explanations of organismal distributions, rather than ascribing each distribution to the vagaries of idiosyncratic evolutionary histories (Humphries & Parenti, 1999).

Soon after, the challenge to vicariance biogeography as a general theory was the commonplace observation that vicariant cladogenesis (i.e., allopatric speciation) is only one of three general macroevolutionary processes, along with dispersal and extinction (MacArthur & Wilson, 1967; Ree & Smith, 2008). Indeed, ecologists have long understood dispersal to be a pervasive process influencing biogeographic distributions (Cowie & Holland, 2006). Long-distance dispersal has been documented in the formation of many biotas worldwide (Sanmartín & Ronquist, 2004; Bacon, Baker & Simmons, 2012) including those in the Neotropics (Smith et al., 2014; Tagliacollo et al., 2015b; Hawlitschek, Ramírez Garrido & Glaw, 2017; Antonelli et al., 2018).

### Inferring landscape evolution in the Neotropics

Neotropical historical biogeography increasingly relies on geological models that specify the landscape configurations on which species originate, disperse, and go extinct. Understanding phylogeny and biogeography in the context of landscape evolution requires assessment of geological data, including sedimentary environments, sedimentation rates, palaeontological records, and geochronological ages, among others (Salo et al., 1986; Räsänen et al., 1995; Räsänen, Salo & Kalliola, 1987; Hoorn et al., 1995; Lundberg et al., 2000; Figueiredo et al., 2009; Hoorn et al., 2010b; Lagomarsino et al., 2016; Sanín et al., 2016; Jaramillo et al., 2017; Hoorn et al., 2017).

Some recent reconstructions of the Neogene landscape in Amazonia are based on dynamic topography, in which mantle movements through time are quantified (Shephard et al., 2010). The effects of these movements are estimated on surface subsidence and are then related to environmental and landscape changes, such as the model applied to explain the genesis of the Pebas wetland in western Amazonia (Hoorn et al., 2010a). Another approach is to use numerical modeling and create reconstructions from physical parameters such as rates of erosion and mountain uplift. An example is the reconstruction of the flow of the Amazon River which incorporates surface processes, flexural isostasy and crustal thickening due to orogeny into a mathematical model to explain the drainage reversal in the Miocene (Sacek, 2014). However, this study did not incorporate the synergic effects of plate movements and surface dynamics, that are known to have impacted on the formation of mega-wetlands and ecosystems (Horton, 2018).

Landscape evolution models (LEMs) can be useful in a biological context but often lack spatial and temporal precision. Biological data can help to infer past landscapes, by testing alternative geological models and increasing their precision. However, we caution that the evolutionary history of one clade might represent an idiosyncratic story, rather than inform the general evolution of an entire landscape in which the clade occurs (Cruz-Neto, Garland & Abe, 2001; Ribas et al., 2012).

In recent years, integrated approaches have built integrative LEMs based on geological, climatic and biodiversity data (Craw et al., 2016; Badgley et al., 2017; Costa et al., 2017). Some studies make use of geographic information systems and combine these with well-dated palynological databases, such as Neotoma (https://www.neotomadb.org/). These models are mainly applied to reconstruct landscapes across Quaternary time scales (i.e., the past 2.6 million years). For example, reconstruction of changes in connectivity across the northern Andes enabled the inference of cyclic phases of biotic dispersal and speciation vs. extinction (Flantua et al., 2015). Molecular phylogenetic data can be used to statistically evaluate the likelihood of competing geological models on longer time scales, such as the closure of the Central American Seaway dividing South and Central America (Bacon, 2013), and the roles of the Caribbean plate margins as dispersal corridors between South and Central America (Tagliacollo et al., 2015a). Similar approaches based on both terrestrial (Baker & Couvreur, 2012) and aquatic taxa (Hrbek, Seckinger & Meyer, 2007) may provide important insights when geological data are insufficient or ambiguous.

### The impact of the Andean uplift on Neotropical diversification

**Figure 3:**
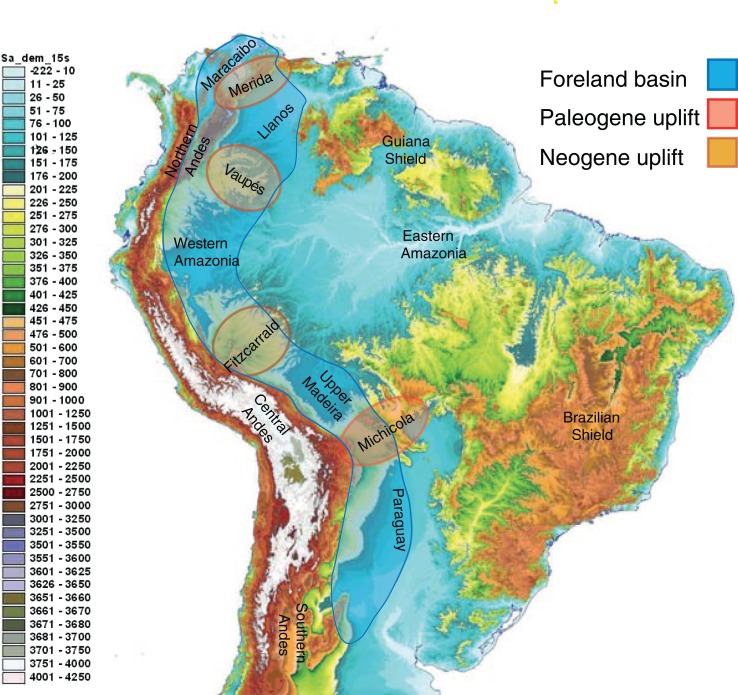
The complex topography and geology of South America. This map highlights the topographic differences across the continent, including the Precambrian and Paleozoic upland shields, and the Andean cordilleras and structural arches that uplifted during the Cretaceous and Cenozoic. The Sub-Andean foreland basin constituted the main drainage axis of South America for most of the past 100 million years, serving as the main arena of evolutionary diversification for the mega-diverse biota of lowland Amazonia. Uplift of structural arches during the Paleogene and Neogene resulted in the formation of the modern continental drainage configuration. Base map created by Paulo Petry from the Shuttle Radar Topography Mission with elevations in meters. Note that the scale exaggerates differences at lower elevations. Adapted from Albert, Petry & Reis (2011).

Neotropical biodiversity can only be properly understood when considering the Andean uplift and the effects of this orogeny on the landscape (Fig. 3) and regional climate (Räsänen, Salo & Kalliola, 1987; Gentry, 1982; Hoorn et al., 2010b). Although the Andes are entirely confined to South America, their formation has led to far-reaching effects across the Neotropics, and there are clear links with orogenies in Mesoamerica caused by plate tectonics.

#### The Andes today

The 7,000 km long Andes is positioned perpendicular to the principal global atmospheric currents and traps the humid air of the Intertropical Convergence Zone. This configuration enhances precipitation along the Andean slopes and in western Amazonia, making them wetter than they would be in a low Andes setting. Moreover, the high Andes also redirects the atmospheric flow inducing the southward deflected South American low-level jet (Garreaud & Muñoz, 2005; Insel, Poulsen & Ehlers, 2010; Rohrmann et al., 2016).

The situation is reversed in southern and in northwestern South America. In these regions, the Andes trap the humid air of the Southern Hemisphere westerlies (Garreaud & Muñoz, 2005). In contrast to the Amazonian settings, the eastern margin of the Andes at its northern and southern extremes forms a rain shadow where semi-desert conditions prevail, and on the western flank there is increased precipitation with more humid conditions (Blisniuk et al., 2005; Palazzesi et al., 2014), although this situation is partially reverse during El Niño events. The monumental Andean barrier has thus imposed strong impacts on both the climate and landscapes of South American lowlands, resulting in the modification of river systems and drastic changes in the climate and habitats of many Neotropical regions.

#### Impact on biotic diversification

The rise of the northern Andes had a major impact on Neotropical biodiversity, as documented for many taxa (Cruz-Neto, Garland & Abe, 2001; Hughes & Eastwood, 2006; Santos et al., 2009; Tagliacollo et al., 2015b; Sanín et al., 2016; Chazot et al., 2016; Diazgranados & Barber, 2017; Bacon et al., 2018). Recent studies that explicitly integrate surface uplift and climatic changes as a function of speciation and extinction include work on the Andean bellflowers (Lagomarsino et al., 2016), Neotropical orchids (Pérez-Escobar et al., 2017), and Neotropical hummingbirds (Condamine et al., 2018).

The Andean uplift affected Neotropical regions in different ways. Over the course of the Miocene, it led to a humidification of Amazonia and aridification of Patagonia (Cione et al., 2005; Blisniuk et al., 2005; Palazzesi et al., 2014; Rohrmann et al., 2016). This contrast is reflected by the history of New World monkeys (Platyrrhini), whose geographic expansion and morphological and taxonomic diversification is tightly linked with climatic changes (Silvestro et al., in press). Platyrrhines were once widely distributed in Patagonia from early to middle Miocene, including the southernmost non-human primates that have ever lived (Tejedor et al., 2006; Novo et al., 2017). However, those primates were later extirpated during regional aridification and global cooling after the Middle Miocene. The platyrrhine record of the high Chilean Andes indicates that the connection between Patagonia and the northern Neotropics possibly persisted on the western part of South America, as the southern Andean cordillera was not an important barrier in the Middle Miocene (Flynn et al., 1995). This scenario provided primates and other animals with a migration route to the north, facilitating faunal turnover (Tejedor & Muñoz-Saba, 2013). This connection may also have contributed to the subsequent Amazonian diversification of crown platyrrhines, including some Patagonian lineages (Rosenberger et al., 2009).

Advances on climatic reconstructions via historical records and climatic modeling (Cheng et al., 2013; Wang et al., 2017) or biome palaeo-distribution modeling (Carnaval & Moritz, 2008; Werneck et al., 2011, 2012a; Ledo & Colli, 2017) allow for direct hypothesis testing based on independent biodiversity data.

## The Four Scales of Biodiversity Research

**Figure 4:**
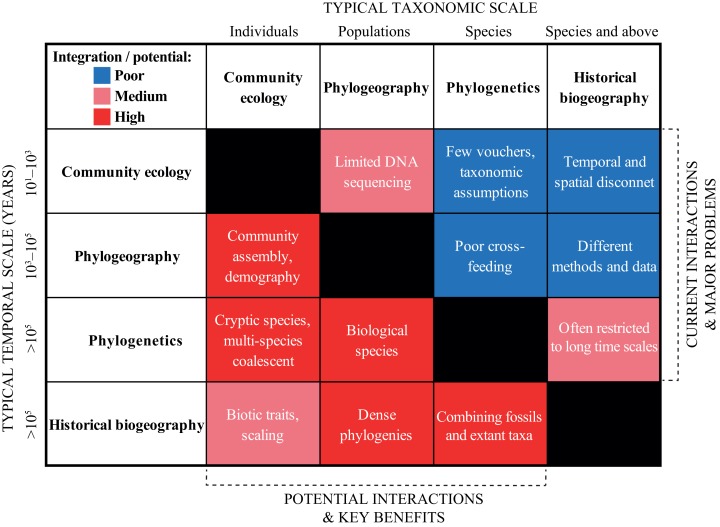
Heat map summarizing the current (right upper boxes) and potential (left bottom boxes) interactions across the biodiversity disciplines in the Neotropics. The *X-* and *Y*-axes indicate the typical taxonomic and temporal scales covered by each discipline, respectively. The white text in each box provides some short examples of why the disciplines are not yet successfully integrated, and some of the key benefits that will be gained by a further integration. See text for a discussion.

In the previous section, we urged for a broader integration *across* the scientific disciplines. We exemplified “trans-disciplinary biogeography” by showcasing the strong links that exist, for instance, between geological and biological fields. Now, we wish to deepen the discussion *within* biodiversity research in a more traditional sense. We do this by discussing and contrasting the current and potential levels of interaction across four sub-disciplines that span the taxonomic, temporal and spatial scales: (1) community ecology, (2) phylogeography, (3) phylogenetics, and (4) historical biogeography (Fig. 4).

## Community Ecology

A major question in the study of biodiversity in general, and Neotropical research in particular, is how ecological communities have been assembled over time and how abiotic factors and species interactions have influenced this process. Approaches for the study of biodiverse communities have employed a wide range of models with diverse conceptual roots. Over the last 20 years, there has been an expansion from studies focusing on contemporary community structure and spatial patterns of physical properties of ecosystems (Tuomisto, Ruokolainen & Yli-Halla, 2003; Heithaus, 1979; Gentry, 1988; Duellman, 1989; Tuomisto et al., 1995; Kalko & Handley, 2001; Garnier et al., 2004), to studies focusing on historical aspects of community structure and evolution (Leite & Rogers, 2013; Smith et al., 2017). Early approaches (Margalef, 1963) focused on indices of diversity, descriptions of community membership, as well as flow charts of energy and nutrients through the community. Key variables influencing community structure consistently emphasized classic Hutchinsonian processes such as resource use, competition, and niche partitioning. However, as ecologists adopted new techniques, the resolution of the niche increased from simple variables to also include high-resolution data on climate, soil chemistry, microbiomes, and other physical and biological properties. These approaches have guided several recent analyses of tropical groups, including microorganisms and plants (Tuomisto et al., 2003; Costa et al., 2009a; Mendes et al., 2015; Arellano et al., 2016; Tuomisto et al., 2016). Additionally, the availability of spatially explicit online global datasets of climate and environmental parameters has helped spawn a generation of studies using large-scale spatial biodiversity surveys and inventories. When analyzed with statistical approaches, these data allow the interpolation between sampled sites and estimation of diversity in non-sampled areas (Costa et al., 2007, 2009b; Ter Steege et al., 2011, 2013).

### Niche-based studies

The general idea that species are adapted to their environment (i.e., have different niches) has two important consequences. First, species distributions are expected to reflect the distribution of suitable habitats. Second, species composition in local communities should reflect the environmental characteristics of the site, as unsuitable environmental characteristics or biotic interactions make it impossible for a species to establish and/or survive. Along these lines, many studies have aimed to characterize the edaphic associations of tropical plant species (Tuomisto & Poulsen, 1996; Phillips et al., 2003; Costa, Magnusson & Luizao, 2005; Roncal, 2006; Zuquim et al., 2009; Kristiansen et al., 2012; Tuomisto et al., 2016; Cámara-Leret et al., 2017; Figueiredo et al., 2017) and the elevational ranges of many taxa (Kluge, Bach & Kessler, 2008). If there are more species adapted to some environmental conditions than others and dispersal is generally not a limiting factor, a species richness gradient should result. However, it is also possible that some environmental conditions may allow more species to coexist than others. Several studies have analyzed species richness gradients along environmental gradients such as elevation (Kluge, Kessler & Dunn, 2006; Brehm, Colwell & Kluge, 2007), rainfall (Clinebell et al., 1995; Esquivel-Muelbert et al., 2017), and soil fertility (Costa, Magnusson & Luizao, 2005; Ter Steege et al., 2006; Tuomisto, Zuquim & Cárdenas, 2014). In general, these studies have shown that Neotropical plant species richness tends to be highest in warm, humid, and aseasonal environmental niches at low to middle elevations.

### Neutral and non-neutral perspectives

In contrast to niche-based processes, spatial patterns in the abundance of anurans from Central Amazonia have been shown to conform to the expectations of Hubbell’s neutral theory of biodiversity and biogeography (NTBB; Hubbell, 2001; Diniz-Filho et al., 2011). More recently, a study demonstrated that the incorporation of population genetic dynamics into NTBB support the hypothesis that biodiversity dynamics are out of equilibrium (Manceau, Lambert & Morlon, 2015). Additional research is needed to assess the relative roles of niche constraints, neutral, and non-neutral processes, in explaining and predicting Neotropical biodiversity.

### Ecological interactions

Early theoretical ecologists conceived the role of ecological interactions (e.g., hervivory, pollination, frugivory) in shaping natural communities, mainly through mechanisms of competition and predation (Hazen, 1964; Boucher, 1988). Later, theoretical ecology shifted to a broader perspective, when facilitation (i.e., positive interactions such as mutualism and symbiosis) was envisioned as a mechanism that affects processes in both population and community levels (Bruno, Stachowicz & Bertness, 2003). This broad spectrum linked many ecological concepts and type of data (such as geo-referenced occurrences and DNA sequences), creating a multi-layer framework for investigating macro-evolutionary processes and patterns.

The use of multi-layered data have shed light on the role of climate gradients in pollinator turnover (Correa Restrepo et al., 2016), the role of frugivory traits in palm diversification (Onstein et al., 2017) and demographic and spatio-temporal distribution of species interactions (Beck, 2006). Coupling time-calibrated phylogenetic and ecological data of ant-plant interactions in the Neotropics also allowed the reconstruction of the geographical origin of the Acacia-ant interaction (Gómez-Acevedo et al., 2010) and the identification of ecological and macro-evolutionary patterns in ant symbioses (Chomicki, Ward & Renner, 2015). In addition, phylogenetic and network analyses disclosed that specialized pollination interactions can display asymmetrically dependent diversification (Ramírez et al., 2011), revealing that specialized interactions might dilute the ecological signal in macro-evolutionary processes. Furthermore, comparative phylogenetic analyses using multi-layered data suggest that mutualistic interactions drive the relative higher diversification rates of frugivorous bats in some Neotropical regions (Rojas et al., 2012) and highlight the putative role of bat seed dispersal in shaping species-rich meta-communities.

## Phylogeography

Phylogeographic research based on dense geographic and molecular sampling at the intraspecific level (subspecies and populations) has advanced significantly in the Neotropics. Specifically, phylogeographic studies of widespread species has improved our understanding of genetic diversification across various biomes. It might be intuitive to assume that species are confined to particular biomes, so that most phylogeographic studies should be done at such spatial scale, but this might not necessarily be the case. We addressed this possibility by synthesizing the recent data presented in Antonelli et al. (2018), where all available Neotropical species in six major clades were codified as present or absent in ten broadly defined biomes or regions: Andean Grasslands, Amazonia, Atlantic Forests, Caatinga, Cerrado and Chaco, Dry Northern South America, Dry Western South America, Mesoamerica, Patagonian Steppe, and West Indies. Our compilation shows that a substantial proportion of species in each clade occurred in more than a single biome or region: angiosperms (27,875 species or 36% of the total number of species analyzed), frogs (232 spp., 17%), birds (1,440 spp., 43%), ferns (1,529 spp., 40%), mammals (530 spp., 42%), and squamates (482 spp., 23%). Clearly, cross-biome transitions have taken place at various taxonomic levels, including populations and species.

Examples of phylogeographic studies across biomes include investigations of major vegetation transitions between Amazonia and the Cerrado (Gehara et al., 2014; Melo et al., 2018) and across biomes with closer functional affinities, such as those of the dry diagonal (Werneck et al., 2012a). Another recent study revealed phylogeographic patterns of disjunctly distributed taxa, which led to inferences on the past connectivity among biomes (Thomé et al., 2016; Prates et al., 2016b, 2016a). It should be noted, however, that at least some of the species of apparently widespread distribution across multiple biomes may in fact not have left their preferred habitat—such as Amazonian species being found along gallery forests in the Cerrado (see Discussion in Antonelli et al., 2018).

Comparative phylogeographic approaches can lead to robust inferences of lineage diversification, and even challenge traditional allopatric scenarios, as has been shown for Neotropical rainforest birds (Smith et al., 2014), or for the synchronous demographic expansion detected for the xeric Caatinga herpetofauna (Gehara et al., 2017). In the Neotropics, hierarchical approximate Bayesian computation analyses (Hickerson, Stahl & Lessios, 2006) have been a popular option to reconstruct patterns of shared phylogeographic history (Carnaval et al., 2009; Prates et al., 2016a; Gehara et al., 2017). More recently, a paradigm shift has been proposed for the field of phylogeography, arguing for a focus placed on trait-based hypothesis testing rather than the more traditional approach of concordance amongst taxa (Papadopoulou & Knowles, 2016). Although this is a relatively new advance in the field, it may gain popularity, considering the general lack of concordance in the distribution of many taxa and the possible range of underlying causes. For instance, a recent study of Neotropical fishes showed that population structure is not concordant amongst palaeodrainages, but rather reflects the fundamental differences in riverine history (Thomaz, Malabarba & Knowles, 2017).

## Phylogenetics

The explosion of molecular phylogenetics and dating analyses allow the inference of time-calibrated trees, where branch lengths are measured as units of time or rates of molecular evolution. Fortunately, a massive increase in the availability of genetic information is being driven by high throughput sequencing technologies. Novel genomic data are likely to significantly improve our understanding of genetic diversity and evolutionary relationships among and within species (Jin & Chakraborty, 1994). Furthermore, these data will also greatly improve our understanding of largely under-studied groups, such as soil microbes (Mahé et al., 2017).

The integration of time-calibrated trees into phylogeographic and biogeographic analyses now enables the establishment of links with external sources of temporal information such as landscape evolution, geological history, fossil record, and climate history. Therefore, phylogenies constitute the strongest and most concrete “bridge” across scales and disciplines as outlined here (Fig. 4). Given the complexity and challenges with the reconstruction of reliable phylogenetic trees, we refer to an accompanying review in this volume (Bravo et al., 2018).

## Historical Biogeography

### Single clade approaches

Detailed reconstructions of the temporal and spatial evolution for individual clades are obtained through “single clade” approaches. These approaches focus on contingencies or events that are idiosyncratic to the group under study, instead of generalities across groups. Methodological advances in single lineage approaches have undergone major developments with parametric methodologies (Ree & Smith, 2008; Lemey et al., 2009; Landis et al., 2013; Matzke, 2014; Landis, 2017; Table 7).

**Table 7:** Methodological challenges and advances for estimating biogeographic histories.

Inferring the spatial and temporal dimensions of evolution are fraught with difficulties, especially due to a lack of abundant and evenly sampled biological and geological data. This is particularly critical for the Neotropics, due to the region’s immense size, relatively limited access, extraordinary biodiversity levels, landscape heterogeneity, and complex evolutionary and geo-climatic histories. To tackle these problems, we summarize some of the main issues associated with the analyses of biogeography and diversification, focusing on how those issues affect the inference of geographic range evolution of lineages in the Neotropics.
**Definition and use of areas for analyses.** Defining units of study in biogeography is not an easy task, especially when diverse systems are involved such as the Neotropics. Sympatry, or the geographic congruence among the distribution areas of taxa, is often used as a criterion to define units for these studies. The identification of such areas has long been based on expert opinion, with data-driven approaches that use actual species distribution data only becoming available more recently (Holt et al., 2013; Vilhena & Antonelli, 2015; Edler et al., 2016; Antonelli, 2017a, 2017b). These approaches to bioregionalization allow for more objective and reproducible analyses. Areas have also been defined using geologically explicit criteria, including information on the geological history of landmasses or geographic barriers, both of which are not exclusive to the group under study (Antonelli et al., 2009; Albert et al., 2011; Töpel et al., 2016; Bacon et al., 2018). Areas defined based on species distribution patterns and geological history are of particular interest (Perret et al., 2007; Givnish et al., 2014; Tagliacollo et al., 2015b).
The use of areas as discrete entities is useful in parametric biogeographic models where areas are considered as traits that evolve along the phylogeny, and whose ancestral areas are estimated at speciation events (nodes). In these models, the spatial units of analysis are defined by the biogeographic hypothesis under examination. For example, it is possible to determine whether diversification rates have been historically higher in Andean or non-Andean taxa (Chazot et al., 2016). However, defining areas as discrete entities is difficult when there are overlapping boundaries and an excess of widespread taxa. Models have been proposed to objectively define areas of endemism by overlapping taxa with “fuzzy” boundaries (Szumik et al., 2002; Szumik & Goloboff, 2004). Similarly, biotic element analyses have also been proposed to test for non-random distributions of species ranges (Hausdorf & Hennig, 2003). Some of these methods have been applied to Neotropical taxa (Casagranda, Roig-Juñet & Szumik, 2009; Guedes, Sawaya & Nogueira, 2014; Noguera-Urbano & Escalante, 2015; Azevedo, Valdujo & Nogueira, 2016).
**Alternatives to the use of areas.** One possibility is to use geographic barriers, rather than areas, as units of analysis, thus focusing on vicariance (Hovenkamp, 1997; Arias, Szumik & Goloboff, 2011; Arias, 2017). This approach explicitly introduces the spatial (landscape) aspect missing from the predefined areas-as-discrete entities used in parametric biogeography. Since this approach is based on taxon-defined ranges, biogeographic reconstructions are not dependent on different area definitions (Arias, 2017). A parametric version of this approach allows geographic (dispersal) barriers to evolve over time within the landscape, something that has been particularly useful for understanding the biogeographic history and evolution of Neotropical freshwater fishes (Albert et al., 2017).
An additional alternative to using discrete areas in biogeographical analyses is the spatial diffusion approach, which conducts spatial-temporal reconstructions under random walk models within likelihood (Lemmon & Lemmon, 2008) or Bayesian (Lemey et al., 2010) frameworks. This approach has been used to study taxa from dry Neotropical biomes (Werneck et al., 2011, 2012a; Nascimento et al., 2013; Camargo et al., 2013), and taxa with broad continental distributions (Gehara et al., 2014). A further development of this approach has been applied to the Neotropical bird genera *Psophia* and *Cinclodes* (Quintero et al., 2015). The method uses georeferenced point-localities to infer ancestral areas and thus does not make assumptions about species ranges and operational units that fit many taxa. On the other hand, this method suffers from the common issue of ancestral lineages occupying average values of the descendant lineages. For instance, analyses with this method have reconstructed the ancestral of *Cinclodes* ovenbirds to a region in-between the western and eastern margins of South America, where no such species occur today (Quintero et al., 2015). Considering the complex and dynamic nature of the Neotropical region, diffusion analyses would certainly benefit from the incorporation of landscape-explicit models that allow the reconstruction of actual paths along branches and fossil-informed diffusion approaches (McRae et al., 2008; Meseguer & Condamine, 2018). These developments would allow the incorporation of spatial heterogeneity via dispersal constraints, derived from estimated ecological niche models or landscape evolution models.
**Estimating geographic evolution on single clades.** Dispersal-extinction-cladogenesis (DEC) is likely the most popular parametric biogeographic method for estimating the geographic evolution of lineages within a particular clade. This likelihood-based method infers anagenetic evolution (i.e., along branch internodes) as a function of two rate parameters: range expansion (dispersal) and range contraction (local extinction). Cladogenetic evolution (i.e., at speciation nodes) is modeled as the likelihood of alternative range inheritance scenarios that describe the division of ancestral ranges into descendant nodes: sympatric speciation or allopatric (vicariance) speciation, and peripheral isolate speciation in the case of widespread ranges (Ree & Smith, 2008).
The popularity of DEC is based on the fact that, given a time tree and associated terminal distributions, it can provide detailed biogeographic reconstructions of the ancestral origin of a clade and the history of dispersal and extinction events that shaped its spatial evolution (Sanmartín & Meseguer, 2016). A potential drawback of DEC is, however, the number of areas that it can implement. A large number of unit areas rapidly leads to computational and convergence issues. Constraining the number of states based on biological or geological criteria is a way to decrease model complexity (Ree & Sanmartín, 2009).
Bay-area, a data augmentation approach based on stochastic mapping that extends the DEC model to deal with a large number of unit areas, has been proposed to tackle the limited number of areas allowed in DEC (Landis et al., 2013). Another extension of DEC is the DEC+J model, which introduces an extra parameter (“J”) to model “jump dispersal” or “founder-event” speciation (Matzke, 2014). The DEC+J model has been used in Neotropical biogeography (Matos-Maraví et al., 2014; Chomicki & Renner, 2015; Espeland et al., 2015), but was recently criticized due to statistical bias (Ree & Sanmartín, 2018). Recently, a new extension was introduced to allow for a time-heterogeneous dispersal process in a Bayesian framework, the “epoch model” (Landis, 2017). This model can be used in the biogeographic dating of speciation events when no fossil or other calibration method exists, and was recently applied to Neotropical cycads (Said Gutiérrez-Ortega et al., 2017).

Typical biogeographic analyses use time-trees and parametric models of biogeographic evolution to estimate ancestral ranges of lineages (branches) and speciation events (nodes), and to infer rates of biogeographic processes (e.g., dispersal, speciation, and extinction). To date, probably hundreds of studies have examined the biogeographic history of particular Neotropical clades in this way. Biogeographic hypotheses or models about the relative role of biogeographic processes in the geographic evolution of particular groups can be compared statistically using methods for model selection in phylogenetics, such as the Akaike information criterion or Bayes factors (Bozdogan, 1987). Moreover, the rates of these processes may be modified (scaled) to reflect the changing connectivity among the areas of analysis over time (Ree & Smith, 2008). These advances have contributed to the integration of landscape dynamics and geological history into taxon biogeography in the Neotropics (Givnish et al., 2014; Palazzesi et al., 2014; Tagliacollo et al., 2015b; Chazot et al., 2016).

### Cross-taxonomic (multi-clade) approaches

These approaches (sometimes under the umbrella of “comparative biogeography”; Antonelli, 2017b) aim to extract generalities on the evolution of a biogeographic region or whole biota, or generalities on the relationships among biogeographic regions or biotas, by reconstructing the history of their individual components. The focus of this approach is on inferring shared biogeographic histories, such as general patterns of colonization and diversification or a common response to climatic and landscape changes. A recent cross-taxonomic analysis for six major clades of terrestrial plants and animals, across all major Neotropical biomes and regions, was presented by Antonelli et al. (2018). That study showed an unexpectedly high number of dispersal events across the entire Neotropics, which took place for tens of millions of years and often involved shifts in major environmental types (in particular from forests to savannas). The high frequency of dispersal events identified in that study reflects patterns reported for tree communities across Neotropical rain forests (Dexter et al., 2017) and between rainforests and savannas (Simon et al., 2009).

Multi-clade approaches were traditionally known as “area biogeography” and were the focus of the cladistic biogeographic school for decades (Nelson & Platnick, 1980; Humphries & Parenti, 1999). The first methods used for cross-taxonomic biogeographic approaches were based on parsimony, which does not allow the formal integration of a temporal dimension (Crisci et al., 1991; Marshall & Liebherr, 2000; Sanmartín, 2016). The incorporation of time into event-based methods then allowed the identification of dispersal corridors and barriers, such as those underlying the assembly of freshwater fish faunas in South American river basins (Dagosta & De Pinna, 2017). Recently developed parametric approaches (Ronquist & Sanmartín, 2011; Sanmartín, 2016) offer now a powerful way to obtain generalities about patterns of dispersal and diversification in biotas, allowing us to test between alternative geological or spatial scenarios (Sanmartín, Van Der Mark & Ronquist, 2008). An interesting methodology bridging community ecology and cross-taxonomic biogeographic analysis is the phylogeographic concordance factor analysis (Satler, Zellmer & Carstens, 2016), which uses Bayesian concordance analysis (Ané et al., 2007) to test for shared evolutionary history among co-distributed species and the existence of strong ecological interactions or dependence (Satler & Carstens, 2016, 2017).

## Bridging the Classical Biodiversity Disciplines

Previous sections described the complexity of Neotropical biodiversity, outlined major knowns and unknowns, proposed a general integrative framework, and discussed approaches and applications of methods depending on the scale. Here, we provide a few examples of how to expand beyond the traditional boundaries and scales of the disciplines related to those in Fig. 4.

### Assembling biodiversity: from communities to biotas

The Theory of Island Biogeography (TIB; MacArthur & Wilson, 1967) introduced parameters such as rates of colonization (immigration) and extinction within a mathematical framework, allowing the prediction of the number of species present on an island based solely on its distance from a mainland species source and its area (Losos & Ricklefs, 2009; Warren et al., 2015). New models inspired by the TIB are now attempting to integrate additional parameters, such as speciation and island age (Whittaker, Triantis & Ladle, 2008), population abundances (Rosindell & Harmon, 2013), and trophic interactions (Gravel et al., 2011).

Community ecology and an expanded TIB are now also adopting a more evolutionary approach by integrating phylogenetic data to the study of community assembly and dynamics, including the role of in situ adaptation or speciation vs. dispersal in community assembly, the temporal sequence of species interactions, and the role of abiotic and biotic factors in diversification of specific lineages (Webb et al., 2003; Sanmartín, Van Der Mark & Ronquist, 2008; Kursar et al., 2009; Valente, Etienne & Phillimore, 2014; Valente, Phillimore & Etienne, 2015, 2018; Cabral, Valente & Hartig, 2017). Importantly, this requires denser voucher sampling of specimens, which in turn will lead to denser phylogenies, better estimates of species boundaries under multi-species coalescent approaches and tackling the common problem of cryptic species (Fig. 4; Bravo et al., 2018). By adopting a more historical focus, community ecology methods are explicitly trying to reconstruct the sequence of events leading to modern-day communities, such as the island-like patches of white-sand savannas in Amazonia (Alonso, Metz & Fine 2013). These approaches relax the assumption of ecological neutrality, and focus on the distinctive properties of individual lineages, historical contingency, and particularities of present-day outcomes (Emerson & Kolm, 2005; Sanmartín, Van Der Mark & Ronquist, 2008). In their most recent forms, these models incorporate ecological parameters such as competition and species interactions (Clarke, Thomas & Freckleton, 2016) or landscape dynamics (Aguilée, Claessen & Lambert, 2013).

### Scaling up community ecology approaches

**Figure 5:**
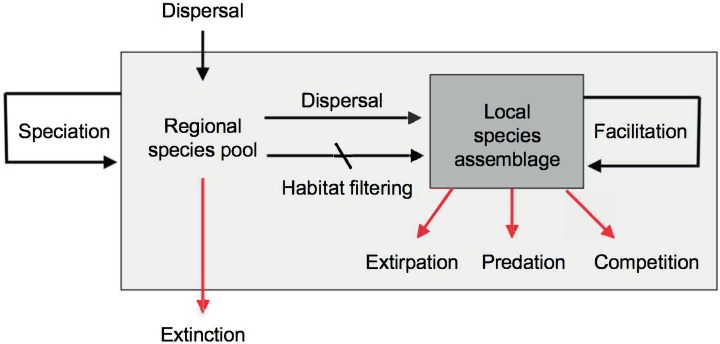
Main evolutionary and ecological processes contributing to the formation of species richness. The regional species pool (light gray box) is defined as the sum of all the local species assemblages (darker gray box). Black arrows indicate processes that increase species richness, red arrows processes that reduce species richness. Note the hierarchical organization of processes resulting in species richness, with evolutionary processes occurring over regional to continental spatiotemporal scales and ecological processes occurring over local scales. Speciation and dispersal contribute new species to the regional pool, while extinction removes species. Dispersal mediated by abiotic habitat filtering and biotic facilitation (Kraft & Ackerly, 2014) increase the richness of local assemblages by enhancing establishment of species preadapted to local conditions, or aiding in the establishment of other species. Biotic interactions such as predation and competition may serve to reduce local richness. Diagram modified from Schluter & Ricklefs (1993) and Albert, Val & Hoorn (in press).

The original goals of community ecology, as established in the early 20th century, were to predict species distributions and abundances, species richness and equitability, community productivity, food web structure, predator-prey dynamics, succession, and community assembly. However, this discipline has not yet succeeded in meeting most of these goals (Ricklefs, 2008; Ritchie, 2009; Vellend, 2010; Ricklefs & Jenkins, 2011; Weber & Strauss, 2016). The reasons are many, but may be especially associated to the non-equilibrium condition of most local assemblages, in which the effects of historical contingencies of dispersal, extirpations, and other stochastic processes override the equilibrium expectations generated by local functional processes such as predation and competition (Fig. 5). In other words, the species composition and equitability of most local assemblages are more strongly governed by regional and historical factors than by local ecological interactions (Mittelbach & Schemske, 2015; Manceau, Lambert & Morlon, 2015; Fukami, 2015; Weeks, Claramunt & Cracraft, 2016).

This “crisis” in community ecology has fueled the rise of alternative functionally-neutral theories, like the NTBB (Hubbell, 2001), and the metacommunity theory (Leibold et al., 2004). However, neutral theories have also been criticized for their simplistic assumptions and lack of predictive power under the non-neutral conditions frequently observed in nature (McGill et al., 2006). In general, the field of community ecology appears to be ripe for a paradigm shift (DeAngelis & Grimm, 2017).

While many studies conducted at continental to global scales aim to test broad hypotheses about drivers of biodiversity gradients (Tuomisto, Zuquim & Cárdenas, 2014; Fine, 2015), others rely on analyses of region-wide field data collected over decades (Ter Steege et al., 2013). These surveys set the stage for analyses on the environmental and historical correlates of diversity (Benavides et al., 2005; Stropp, Ter Steege & Malhi, 2009; Ter Steege et al., 2013). Detailed explanations of the heterogeneity found at multiple scales remain a major challenge.

One recent topic of concern is whether Neotropical biodiversity patterns documented today have resulted from purely “natural” processes, or have been largely influenced by human activities (Levis et al., 2017). Evidence from archaeological, remote sensing, biodiversity data, and modeling approaches suggest that humans may have had a much deeper impact on Neotropical biodiversity, both in time and space, than traditionally conceived (Table 8).

**Table 8:** Human impacts on Neotropical biodiversity.

Humans have occupied the Neotropics since about the end of the Late Pleistocene (10–20 kya) and were likely instrumental in promoting the extinction of the diverse fauna of large-bodied mammals (Sandom et al., 2014). The drastic decrease in the density and diversity of large mammals also resulted in major changes to the overall vegetation structure (Bond, 2005). For example, in South America, the limits between the dry diagonal and the adjacent forests might have shifted significantly compared to where they would have been without any human involvement and its cascading effects (Doughty, Faurby & Svenning, 2016). In addition to anthropogenic extinctions, humans might also have caused drastic range contractions of many other species, and reduced the abundances of others (Faurby & Svenning, 2015). The human-linked reduction of the Neotropical megafauna may also have affected the plants that these animals dispersed. This pattern was recently discussed in the context of the impact of over-hunting of primates and tapirs on the total woody biomass of Amazonia (Peres et al., 2016), and large frugivorous mammals in the Atlantic forest (Bello et al., 2015). Overall, it seems that the patterns observed could reflect the pressures of overhunting in pre-historic times (before 500 years ago). Humans have restricted the ranges of some species, but actively or passively expanded the ranges of others, such as invasives or domesticated species (Levis et al., 2017, 2018). Knowledge to date is based on the best-studied groups and it remains unclear whether substantial effects of humans might be frequent among other organisms. We anticipate that this will become an active area of research for the coming years.
Apart from the effects of past human activity on Neotropical biodiversity, current habitat loss, climate change and neglected conservation strategies pose increasingly serious threats to natural landscapes. Indeed, these are widely known to be the primary drivers of the current global biodiversity crisis. Studies that quantify genetic diversity, vulnerability, and extinction risk derived from the impact of habitat loss and climate change are essential to grasp how current human activities are expected to impact the future of Neotropical diversity at multiple levels. Although we now have a fair understanding of several components of Neotropical biodiversity, for many taxonomic groups, well-defined processes remain elusive and biases loom large; refining these issues will constitute an area of active scientific exploration for the next decade and beyond.

### Exploring the tripod: ecological interactions-macroevolution-biogeography

The multi-layer analytical framework developed by recent eco-genetic research granted the combination of multiple data layers from three different scale dynamics (local mechanisms, macro-evolutionary processes, geographic patterns). The synergy of those layers illustrates a tripod that gathers ecological, evolutionary and biogeographical factors of populations, communities, and meta-communities, respectively (Ricklefs & Jenkins, 2011; Hanson et al., 2012; Connolly et al., 2017). Detecting ecological signals across multi-layered data, such as the contribution of mutualisms in biogeographic processes (speciation, extinction, migration) remains a major challenge. Tackling this challenge will require linking spatio-temporal data with models that detect common signatures of ecological interactions across layers (Pilosof et al., 2017). Although recent theoretical advances have unveiled phylogenetic signals from community processes (Rezende, Jordano & Bascompte, 2007; Minoarivelo et al., 2014; Bastazini, 2017), we urge for new models that can identify ecological signal from multiple layers. The exploration of ecological factors that are associated to positive and negative interactions (i.e., network structure, taxonomic associations) might reveal important insights on the dynamics and complexity of ecological interactions for producing and maintaining Neotropical biodiversity.

### Incorporating fossils into biogeography

One important shortcoming of molecular-based biogeographical analyses in general, and parametric models of range evolution in particular, is the fact that it is almost always based on extant data alone. Because of the effects of extinction, the pattern of geographic distribution we observe today may be a poor representation of the actual biogeographic history, especially if extinction rates have been unequal among areas (Sanmartín & Meseguer, 2016) and taxa (Silvestro et al., 2016). One way to solve this issue is to include extinct lineages in biogeographic analyses (Mao et al., 2012), or to use their past distribution inferred from the fossil record to constrain inferences of ancestral ranges (Meseguer et al., 2015). This approach has often revealed different biogeographic histories for the study group as compared to analyses based on extant data only (Mao et al., 2012; Meseguer et al., 2015). A recent development is the development of the dispersal-extinction-sampling model, to infer rates of dispersal and area extinction exclusively from fossil data (Silvestro et al., 2014, 2015). Under this approach, a separate sampling parameter is used to account for the unevenness of the fossil record both spatially and temporally. When the fossil record is sufficiently abundant, it provides more accurate measures of changes in rates of geographic evolution and less biased extinction rates, than when exclusively extant taxa are used (Silvestro et al., 2015).

Another challenge to understanding current patterns of evolutionary diversity is the absolute dating of phylogenies, which relies heavily on fossils. This shortcoming complicates a detailed understanding of the ages of tropical taxa, especially those from rainforests (Wing et al., 2009). New methodological developments to directly integrate fossil (extinct) lineages into phylogeny reconstruction (Ronquist et al., 2012; Heath, Huelsenbeck & Stadler, 2014; Zhang et al., 2016; Silvestro et al., 2016) offer new hope in the quest to retrieve more accurate depictions of evolutionary patterns.

Finally, estimating the tempo of diversification is difficult without fossil constraints. In simulated phylogenies, the resulting shape of lineage-through-time plots vary significantly when the fossil record is added as compared to phylogenies that incorporate extant taxa exclusively (Matos-Maraví, 2016; Sanmartín & Meseguer, 2016). The inferred macroevolutionary dynamics estimated from molecular phylogenies may thus be misleading if fossil taxa are neglected, or when macroevolutionary tools do not acknowledge the rare sampling of fossil lineages. Clearly, fossils are crucial to not only understand past dynamics, but also for an improved understanding of current patterns (Fritz et al., 2013). We therefore urge for a much tighter integration between the palaeontological and neontological research communities in the Neotropics.

### Integrating landscape evolution models into biotic diversification

A potential problem with single clade and cross-taxonomic biogeographic analyses as discussed above is that areas are treated as traits of organisms evolving along phylogenetic trees. Geology is often used to inform the model but does not form its core. For instance, area connectivity is often used in parametric methods to constrain or scale migration rates but not as an actual part of the model. A new generation of methods that use the power of LEMs to study the full panoply of evolutionary processes, at both microevolutionary (population) level (Byrne & Hopper, 2008; McRae et al., 2008) and macroevolutionary (interspecific) scales (Tagliacollo et al., 2015b; Badgley et al., 2017) are now being developed. For example, uplift of a dissected landscape and river capture are two landscape evolution processes with great power to generate high species richness (Albert et al., 2018). Both of these processes simultaneously and continuously merge and separate portions of adjacent landscape areas, allowing biotic dispersal and larger geographic ranges, vicariant speciation and smaller geographic ranges, and extinction when range sizes are subdivided below a minimum persistence threshold (Albert et al., 2017).

## Conclusions

### The origins of Neotropical biodiversity

There are often mixed definitions and questions related to the timing and mode of biotic evolution in the Neotropics. The “origin of Neotropical biodiversity” encapsulates at least two contrasting subjects: the timing of origin of hyperdiversity (i.e., “when did the Neotropics reach globally outstanding levels of species richness?”), and the actual age of extant species (i.e., “when did the species that we see today split from their most recent common ancestors, as defined by their stem age?”; Hoorn et al., 2011). It is clear that there have been unusual periods of time throughout geological history, both in terms of biotic and abiotic events (Jaramillo, 2006; Hoorn et al., 2010b; Jaramillo et al., 2010). However, all periods of time have contributed to the current biodiversity, if seen in the perspective of the “evolutionary continuum” that bridges the core biodiversity disciplines (Fig. 4).

Examples of studies that have sought for “special” periods of time often come from time-calibrated molecular phylogenies. For instance, butterfly species-pairs seem to be relatively young in origin (i.e., <2 Ma), suggesting that the Pleistocene and Holocene may have represented “extraordinary times” for Neotropical butterfly speciation (Garzón-Orduña, Benetti Longhini & Brower, 2014) and following the refugium theory (Table 4). However, time-calibrated phylogenies may not fully address the potential impact of extinction and species duration (Hoorn et al., 2011). In other words, if we were able to travel back in time to any period and sequence species around us, the odds are that most species alive might also be around two Ma old or less. In addition, the definition of “species” may vary considerably depending on data source (e.g., based on the fossil record and extant populations) and across taxa. Highly structured populations with considerable genetic divergences may be seen as “incipient species” that have not yet completed the speciation process (Craig et al., 2018). Variable species concepts and adequate sampling of extant and extinct taxa represent a serious barrier for our understanding of Neotropical diversification (Tables 1–3 and 5).

### Advancing Neotropical research

Comparative biology has experienced major advancements in the theory and practice of biogeography and molecular phylogenetics during the past decades. However, we still need to increase sampling of organisms drastically in order to advance our knowledge on the patterns and processes underlying Neotropical biodiversity (Feeley, 2015). However, fieldwork in the Neotropics, especially in pristine areas, is time consuming and logistically demanding. Research funding for exploratory inventory projects is also becoming increasingly harder to obtain, despite the fact that highly successful projects (i.e., sequencing the first human genome and creating the Amazon Tree Diversity Network) were initially discovery-driven, rather than focused on testing specific hypotheses. Furthermore, obtaining permits to collect and export biological samples is also challenging, involving many differences across national legislations. Finally, some authors have seen the need of fieldwork as less relevant in this era of museomics (Buerki & Baker, 2016; Zedane et al., 2016).

Despite all these obstacles, fieldwork remains absolutely essential for biodiversity data generation and monitoring (Albert, 2002). Fieldwork also provides students and researchers with a deeper understanding of their study systems, often providing new ideas and questions, while facilitating the establishment of new collaborations, enabling the exchange of knowledge, fueling the development of new methods, and increasing the possibilities of major discoveries (Fleischner et al., 2017). We should seriously consider new strategies for the generation of new biodiversity data, as well as for the syntheses of the already available data.

Multi-taxon field campaigns could provide unique opportunities for intensive sampling, while optimizing resources, bureaucratic, and logistic efforts. However, this vision requires a radical re-thinking and re-organization. We need to provide young generations with the training, tools and resources needed to carry out research on all aspects of biodiversity. We also need to support taxonomic specialists and institutions in order to adequately study, archive and facilitate free access to biological collections and associated data. In addition, we also need to join forces across nations and disciplines for mutual benefit and joint scientific growth. Clearly, these investments would be worthwhile from a global perspective. The future of Neotropical biodiversity research depends on extensive collaborations and coordinated efforts.

## Take-Home Messages

Five main take home messages can be taken from this review, namely:

1. Neotropical biodiversity is exceedingly high, regardless of the axis explored (e.g., taxonomic, phylogenetic, functional, ecosystems);
2. Understanding the origins, evolution, maintenance, and distribution patterns of Neotropical biodiversity is a grand scientific challenge with many remaining unknowns;
3. “Trans-disciplinary biogeography” aims to better integrate the seemingly disparate disciplines required to explore the biotic and abiotic evolution of the Neotropics;
4. Many methodological advances will be required to deal with the increasing wealth of biodiversity data and associated environmental and geological variables;
5. There is an urgent need to fill the many gaps in biodiversity knowledge, including extant and extinct taxa and their interactions. This calls for a “renaissance” for fieldwork.

## Additional Information and Declarations

### Data Availability

The following information was supplied regarding data availability: The research in this literature review did not generate any data or code.
